# Workplace psychosocial resources and risk of cardiovascular disease among employees: a multi-cohort study of 135 669 participants

**DOI:** 10.5271/sjweh.4042

**Published:** 2022-10-29

**Authors:** Tianwei Xu, Reiner Rugulies, Jussi Vahtera, Jaana Pentti, Jimmi Mathisen, Theis Lange, Alice J Clark, Linda L Magnusson Hanson, Hugo Westerlund, Jenni Ervasti, Marianna Virtanen, Mika Kivimäki, Naja H Rod

**Affiliations:** 1 Stress Research Institute, Stockholm University, Stockholm, Sweden.; 2 Department of Public Health, University of Copenhagen, Copenhagen, Denmark.; 3 National Research Centre of the Working Environment, Copenhagen, Denmark.; 4 Department of Psychology, University of Copenhagen, Copenhagen, Denmark.; 5 Department of Public Health, University of Turku, Turku, Finland.; 6 The Centre for Population Health Research, University of Turku, Turku, Finland.; 7 Turku University Hospital, Turku, Finland.; 8 Clinicum, Faculty of Medicine, University of Helsinki, Finland.; 9 Finnish Institute of Occupational Health, Helsinki, Finland.; 10 Novo Nordisk A/S, Søborg, Denmark.; 11 School of Educational Sciences and Psychology, University of Eastern Finland, Joensuu, Finland.; 12 Division of Insurance Medicine, Karolinska Institutet, Stockholm, Sweden.; 13 Department of Epidemiology and Public Health, University College, London, UK.

**Keywords:** collaboration, leadership quality, procedural justice, social support

## Abstract

**Objective:**

In terms of prevention, it is important to determine effects on cardiovascular disease (CVD) when some workplace psychosocial resources are high while others are low. The aim of the study was to assess the prospective relationship between clustering of workplace psychosocial resources and risk of CVD among employees.

**Methods:**

We pooled data from three cohort studies of 135 669 employees (65% women, age 18–65 years and free of CVD) from Denmark, Finland and Sweden. Baseline horizontal resources (culture of collaboration and support from colleagues) and vertical resources (leadership quality and procedural justice) were measured using standard questionnaire items. Incident CVD, including coronary heart and cerebrovascular disease, was ascertained using linked electronic health records. We used latent class analysis to assess clustering (latent classes) of workplace psychosocial resources. Cox proportional hazard models were used to examine the association between these clusters and risk of CVD, adjusting for demographic and employment-related factors and pre-existing physical and mental disorders.

**Results:**

We identified five clusters of workplace psychosocial resources from low on both vertical and horizontal resources (13%) to generally high resources (28%). High horizontal resources were combined with either intermediate [hazard ratio (HR) 0.84, 95% confidence interval (CI) 0.74–0.95] or high (HR 0.88, 95% CI 0.78–1.00) vertical resources were associated with lower risks of CVD compared to those with generally low resources. The association was most prominent for cerebrovascular disease (eg, general high resources: HR 0.80, 95% CI 0.67–0.96).

**Conclusions:**

Individuals with high levels of workplace psychosocial resources across horizontal and vertical dimensions have a lower risk of CVD, particularly cerebrovascular disease.

Early studies on stressful psychosocial working conditions and cardiovascular disease (CVD) risk were published already in the 1960s (1), and subsequent cohort studies have confirmed an association between work stressors and CVD (2). The leading concepts of psychosocial working conditions include the job–demand–control (job strain) model (3–5), the effort–reward imbalance model (6), the organizational justice model (7), and the job–demands–resources model (8). More recently, it has been suggested that a focus on potential health-protective resources at work may also be useful (9). From a CVD prevention perspective, targeting workplace psychosocial resources may complement traditional workplace interventions, such as wellness and exercise programs (10).

High levels of workplace psychosocial resources have been suggested to be linked to a lower risk of mental health problems including depression (11), the metabolic syndrome (12), and type 2 diabetes (13, 14), lower levels of inflammatory markers such as C-reactive protein, interleukin-6 and tumor necrosis factor alpha (13, 15), and lower ambulatory blood pressure (16, 17), which are all CVD risk factors. To date, however, the evidence on potential health benefits of workplace psychosocial resources is inconsistent and scarce. Published studies have mainly investigated coronary heart disease (CHD) but not cerebrovascular disease (CBD), although the latter contributes to 35% of age-standardized CV-related deaths (18). Three studies found that a higher level of workplace psychosocial resources, operationalized as preferable levels of organizational justice (7), leadership quality (19), or workplace social support (20, 21), were associated with a lower risk of incident CHD, but these associations were not observed in an earlier study of 19 565 full-time employed Swedish women (22), and not for CBD (21). None of these previous studies considered the clustering of workplace psychosocial resources, although resources are likely to cluster and through this clustering be differently associated with health outcomes than what would be expected based on their individual effects. The coexistence of workplace psychosocial resources at organizational, leadership, and group levels (ie, different hierarchical domains) may be dynamic (23). These different sources of resources may potentially affect each other and exert synergistic influences on employees’ health (23). Our previous study identified four distinct resource clusters among Finnish public sector employees, and some clusters were more protective of type-2 diabetes than others (14), but it remained unclear whether this workplace resource pattern could be generalized to the wider working population, including private sector employees, or to other health outcomes. The present paper adds new results towards this end. In terms of prevention, it is important to explore and understand the clustering of workplace psychosocial resources and the potential health effects of such clustering across various hierarchical domains in order to develop effective multilevel interventions aimed at creating healthier workplaces.

To address these limitations, we examined the clustering of four key workplace psychosocial resources (ie, culture of collaboration, social support from colleagues, leadership quality and procedural justice) and assessed whether these clusters were associated with the risk of developing CVD (including CHD and CBD) in three cohorts with a total of 135 669 men and women from Denmark, Finland and Sweden. These cohorts included employees from public and private sectors.

## Methods

### Study population

**Figure 1:**
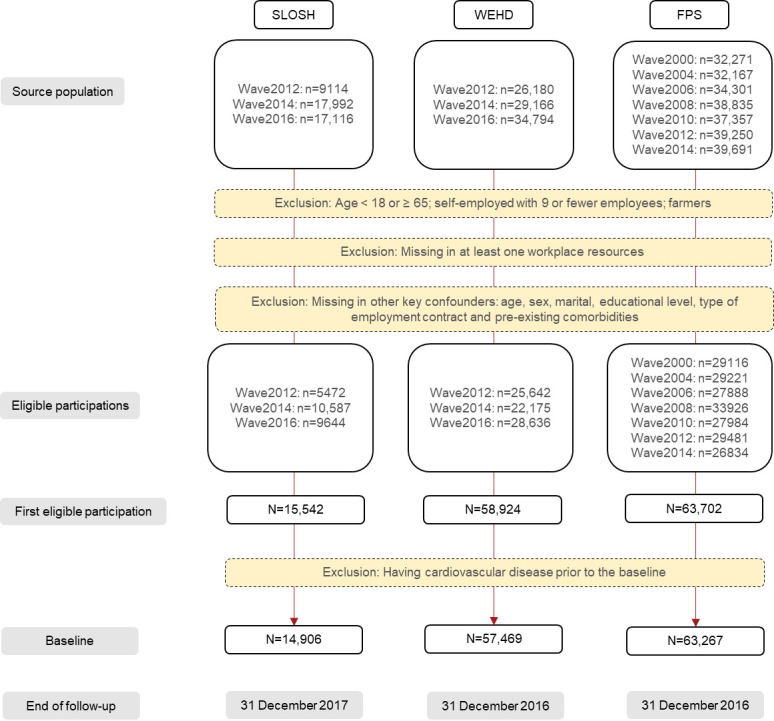
Flow chart of the study population.

We used data from the following three prospective cohort studies: The Work Environment and Health in Denmark (WEHD) study, the Finnish Public Sector (FPS) study and the Swedish Longitudinal Occupational Survey of Health (SLOSH) (figure 1). WEHD is a biennial population-based survey, initiated in 2012 in Denmark, with around 58% respondents working in the private sector (24). FPS is a dynamic cohort of Finnish employees with repeated data collections every two to four years initiated in 1998/2000 onwards (25). FPS consists of employees in the municipal services of ten Finnish town and 21 public hospitals, who had a job contract for a minimum of six months. SLOSH is a population-based cohort initiated in 2006 in Sweden with biennial follow-ups, including 59% participants working in the private sector (26). A more detailed description of these cohorts has been published elsewhere (27).

According to the data availability and to allow cross-wave comparability, we included WEHD waves 2012–2014, FPS waves 2000–2014, and SLOSH waves 2012–2016. Figure 1 depicts the process of baseline establishment, including the exclusion criteria, and end of follow-ups. To ascertain incident CVD during the follow-up, all CVD cases occurred prior to the baseline were excluded (figure 1).

Ethical approval for FPS was obtained from the Ethics Committee of the Hospital District of Helsinki and Uusimaa (25). WEHD was approved by and registered with the Danish Data Protection Agency. Ethical approval was obtained from the Regional Ethical Review Board in Stockholm for SLOSH (26).

### Workplace psychosocial resources

We measured four types of workplace psychosocial resources: (i) culture of collaboration, (ii) support from colleagues, (iii) leadership quality, and (iv) procedural justice to represent hierarchical dimensions of workplace psychosocial resources, ie, group (horizontal), leader (vertical) and organizational (vertical) levels, respectively, using standardized items/scales (supplementary material, www.sjweh.fi/article/4042, table S1) (23). Detailed explanations of choosing the following categorization can be found in supplementary text S1.

Culture of collaboration was dichotomized and good collaboration was defined as the collaborative efforts to achieve the best available results or to develop or apply new ideas in the workplace. The items from the justice and team climate inventories were used as a single item (in WEHD) or as dichotomized by the mean score (in SLOSH and FPS) (28, 29).

Co-worker support on perceived colleagues’ support was dichotomized by whether receiving an affirmative response to one item (from the Danish Psychosocial Work Environment Questionnaire in WEHD (30); from the Demand–Control questionnaire in SLOSH (26); and from Statistics Finland working climate questions in FPS) (31).

Due to the harmonization (14), the leadership variable in FPS and SLOSH included dimensions on caring, listening, appreciative, and informative (three items from The Stress Profile and one item from the relational justice scale) (32, 33). WEHD included slightly different dimensions (8 items including eg, authorization of own work and career development) (24). Leadership quality was categorized into quartiles.

The variable for procedural justice (fairness in the principles and processes leading to decision-making and the distribution of rewards and benefits) was measured using a modified version of Moorman’s scale (34). Procedural justice was categorized into quartiles in FPS and SLOSH. In WEHD, procedural justice was also grouped into four levels (one item) and the highest level of procedural justices was ‘all the time’, followed by ‘often’ or ‘sometimes’, ‘rarely’ and ‘never’.

To understand the heterogeneity of instruments across cohorts, we performed tests for checking the correlations and agreements between the single-item instruments and the full scales (supplementary table S2). There were high correlations (Spearman correlation coefficients >0.8) and moderate to strong levels of agreements (0.60<κ<0.90) between the single-item and scale measures when assessing culture of collaboration. For procedural justice, despite of a high correlation between the single-item measurement (Spearman correlation coefficients >0.8) and the full scale, the agreement was relatively weak (0.40<κ<0.59). Leadership items in WEHD were not identical with those in FPS and SLOSH (the latter two cohorts used exactly same leadership items) and due to lack of shared items, we were not able to perform similar validity analyses as for culture of collaboration and procedural justice.

### Assessment of cardiovascular disease

Using the unique personal identification numbers for each citizen in Denmark, Finland and Sweden, all participants were linked to nationwide health, death and population registers. We used in-patient (all cohorts), out-patient (SLOSH and WEHD) and death (all cohorts) registers to capture incident CVD (ie, cases occurred prior to baseline were excluded). CVD was identified if diagnosed with CHD or CBD. We detected CHD using the main diagnosis codes of ICD-10 I20.0, I20.1, I21–I25 (excluding unspecified angina), and ICD-8/9 410–414, whereas ICD-10 I60-I69 and ICD-8/9 430–438 were used to detect CBD as the main diagnosis. Subtypes of CVD, including myocardial infarction, ischemic stroke and hemorrhagic stroke were also identified using ICD codes (supplementary text S2). Incident CVD events were identified with the earliest diagnosed date after the baseline, after excluding historical CVD events (figure 1).

### Covariates

Confounders were identified prior to data analysis using directed acyclic graphs based on prior knowledge (36).

Key confounders included age, sex, country of birth (Nordic born, other European countries, other continents), educational level (≤9, 10–12, ≥13 years), marital status (unmarried or cohabiting, single, separated or divorced and widowed), type of employment contract (permanent/non-permanent), pre-existing comorbidities (Charlson Comorbidity Score) and pre-existing diagnosed mental disorders (supplementary text S3). These variables were all extracted from the national registers, except that marital status in FPS and employment contract in SLOSH were measured by self-report and there was no information on country of birth in FPS.

Other clinical factors including body mass index (BMI), self-reported mental health and lifestyle factors including smoking (current smoker/non-smoker), risky alcohol consumption (yes/no) and physical inactivity (yes/no) were self-reported at the baseline (supplementary text S3). We considered them to be potential mediators rather than confounders, as they were measured at the same time with the exposures.

### Statistical analysis

We used latent class analysis, a hypothesis-free data-driven approach, to assess clustering of workplace psychosocial resources based on participants’ first eligible participation (36). In previous study based on this approach, we have shown that the latent classes observed at baseline (ie, the first eligible participation) were robust over time and could be extrapolated to all participants regardless of their participation waves (14). In addition to Bayesian Information Criterion (a measurement of model fit), distribution of class membership probabilities, class sizes and interpretability of the classes, we determined the class model according to the comparability across cohorts (supplementary figure S1) (36). Although a four-class solution already showed distinctive patterns in FPS in a previous study (14), using a five-class model with one additional distinctive latent class pattern added in WEHD and SLOSH, the three cohorts were more comparable and we therefore chose to use this five-class solution (supplementary figure S1).

We ran a Cox proportional hazard model with age as the underlying time scale. No violation was detected for proportional hazard assumption (by using log-log plot or by including interaction terms between time and covariates). The model was adjusted for country of birth (when available), marital status, educational level, type of employment contract, and pre-existing mental and physical comorbidity. The incidence rate difference was calculated using the Aalen additive hazard model. Subtype analyses of CHD and CBD were performed. As a supplement, we analyzed each individual type of resources with and without mutual adjustment of the others, using the lowest level of each individual resource as the reference.

We also conducted several sensitivity analyses. In order to reduce the risk that employees who had prevalent, albeit undiagnosed CVD might be more likely to perceive workplace psychosocial resources differently, resulting in reverse causation, a one-year lag-time was applied. We further restricted the follow-up lengths to the first four years, to rule out the impact of differences in follow-up lengths across cohorts. Lastly, models were additionally adjusted for other covariates, ie, lifestyle and clinical factors assumed to be mediators in the primary analysis.

To determine potential effect modification, stratified results were carried out for age groups, by sex and educational levels using the Cox model and the additive hazard model, to estimate interactions on both the multiplicative and additive scales.

We followed a 2-stage approach in which associations were first analyzed in each cohort study separately and then cohort-specific estimates were combined using fixed-effect meta-analysis (R package meta version 4.9-2). We used R package, poLCA version 1.4.1, for latent class analysis, SAS 9.4 procedure, PROC PHREG, for Cox models and R package timereg, version 1.9.3 for additive hazard models. Results from Cox models and additive hazard models were expressed as hazard ratios (HR) and incidence rate difference (IRD), respectively with their 95% confidence intervals (CI). Statistical syntax is provided in the supplementary material).

## Results

### Patterns of workplace psychosocial resources

**Figure 2:**
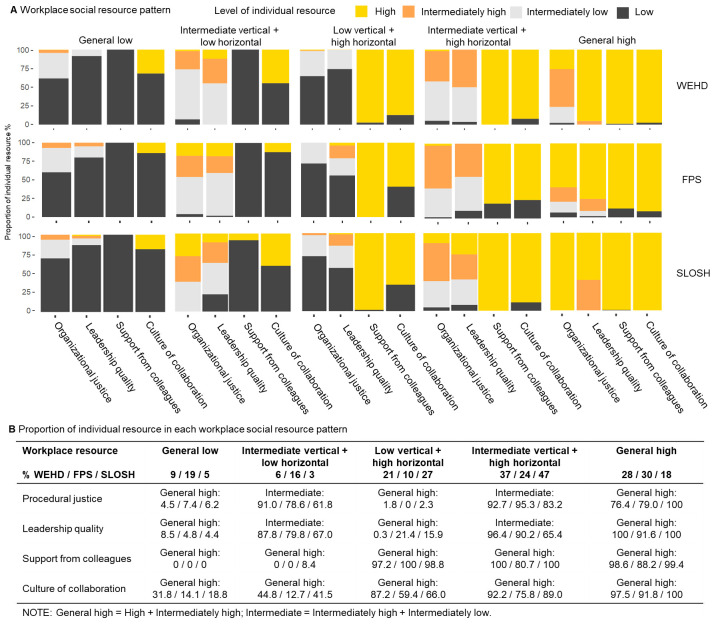
Workplace psychosocial resource pattern in each latent class using data from Work Environment and Health in Denmark study (WEHD: N=57 496), Finnish Public Sector study (FPS: N=63 267) and Swedish Longitudinal Occupational Survey of Health (SLOSH: N=14 906).

We identified five latent classes of workplace psychosocial resources, using 57 496 participants from WEHD, 63 267 from FPS and 14 906 from SLOSH, with similar patterns of workplace psychosocial resources across cohorts (figure 2A). WEHD and SLOSH shared similar distribution of resource clusters, while FPS had a larger proportion of ‘general low’ and ‘intermediate vertical+low horizontal’ and smaller proportion of ‘low vertical+high horizontal’ and ‘intermediate vertical+high horizontal’ resources than WEHD and SLOSH (figure 2B).

Three cohorts together (figure 2B), 13% were categorized into the ‘general low’ class, in which all the four resources were low. The ‘intermediate vertical+low horizontal’ class (11%) consisted of mainly intermediate (i.e. intermediately high and intermediately low) levels of vertical resources (procedural justice and leadership quality), but low levels of social support and culture of collaboration (horizontal resources). The ‘low vertical+high horizontal’ class (17%) was characterized by low level of procedural justice and leadership quality and high levels of social support from colleagues and culture of collaboration. The ‘intermediate vertical+high horizontal’ class (32%) is characterized by an intermediate (i.e. intermediately high and intermediately low) level of procedural justice and leadership quality and a high level of social support from colleagues and culture of collaboration. Lastly, in the ‘general high’ class (28%) individuals reported a relatively high workplace resources across all dimensions. Baseline characteristics are presented in table 1.

**Table 1:** Baseline characteristics of the analytical population by workplace psychosocial resources (N=135 669).

Workplace psychosocial resources	Total (N=135 669)		General low (N=18 040) 13%		Intermediate vertical+low horizontal (N=14 394) 11%		Low vertical+high horizontal (N=22 441) 17%		Intermediate vertical+high horizontal (N=42 900) 32%		General high (N=37 894) 28%	
					
	%	Mean	%	Mean	%	Mean	%	Mean	%	Mean	%	Mean
Demographic characteristics												
Age (years)		44		45		45		45		44		44
Women	65		67		71		61		64		69	
Non-Nordic born ^a^	3		3		4		2		3		3	
Low educational level	24		22		19		30		25		23	
Married	68		70		68		66		68		70	
Clinical characteristics												
Comorbidity score		0.15		0.14		0.13		0.16		0.15		0.15
Body mass index ^b^		25		26		25		26		25		25
Mental disorders	2		2		2		3		2		2	
Lifestyle characteristics												
Current smoker ^b^	19		21		20		21		18		19	
Physical inactivity ^b^	26		32		33		24		25		26	
Excessive alcohol consumption ^b^	11		12		11		12		12		11	
Work-related characteristics												
Temporary job contract	12		11		15		9		12		16	

a Based only on the Work Environment and Health in Denmark (WEHD) study and the Swedish Longitudinal Occupational Survey of Health (SLOSH).

b A total of 36 314 missing existed for these variables, mainly due to the skipped measurement in Finnish Public Sector (FPD) study waves 2000, 2006, 2010.

### Workplace psychosocial resources and cardiovascular disease

**Figure 3:**
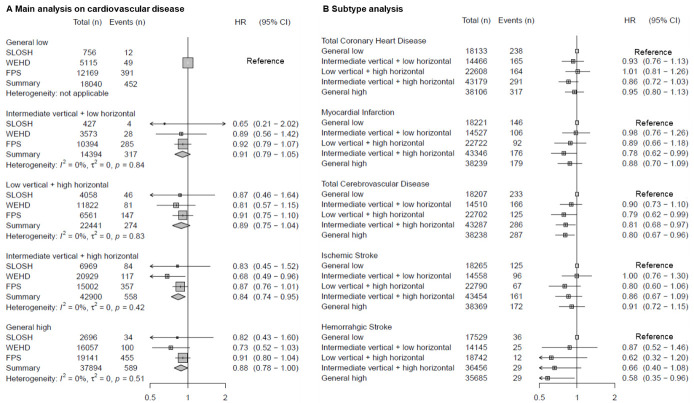
Association between clustering of workplace psychosocial resources and incident cardiovascular disease (CVD), after adjustment for age, sex, country of birth, educational level, marital status, pre-existing comorbidity, pre-existing mental disorders and types of employment contract. Fixed effect meta-analysis.

During a mean follow-up of 6.8 years, 2190 incident CVD cases (26.8 per 10 000 person-years) were recorded among 135 669 initially CVD-free participants (mean age: 44 years, proportion of women: 65%) (table 2). The results across cohorts were generally homogeneous (I^2^<0.1%). Compared to the latent class characterized by low resources (figure 3A), classes with high horizontal resources combined with either intermediate or high vertical resources were at lower risk of developing incident CVD, corresponding to 3.4 (95% CI -6.7– -0.1) and 2.2 (95% CI -5.4–1.0) fewer incident CVD cases per 10 000 person-year, respectively.

**Table 2:** Summary of studies that provided individual participant data used in the analyses for cardiovascular disease, using data from the Work Environment and Health in Denmark (WEHD) study (N=57 496), Finnish Public Sector (FPS) study (N=63 267) and the Swedish Longitudinal Occupational Survey of Health (SLOSH) (N=14 906). [CVD=cardiovascular disease; CHD=coronary heart disease; CBD=cerebrovascular disease.].

Cohorts	Country	Baseline years	Follow-up length (mean, years)	Baseline age (mean, years)	Women,%	CVD^a^	CHD^a^	CBD^a^
WEHD	Denmark	2012–2016	2.0	46	54	32.3	17.4	15.3
FPS	Finland	2000–2014	11.8	43	77	22.0	11.5	11.2
SLOSH	Sweden	2012–2016	4.0	49	59	30.2	18.6	12.7
All	2000–2016	6.8	44	65	26.8	14.7	12.8

a Incidence rate per 10 000 person-years.

Subtype analysis (figure 3B) of 1175 CHD and 1097 CBD incident cases showed the three clusters with high horizontal resources were associated with a lower risk of total CBD, especially with hemorrhagic stroke, but not with total CHD. We observed a lower risk of incident myocardial infarction when perceiving ‘intermediate vertical+high horizontal’ resource.

Excluding cases during the first year or restricting to the first four-year follow-up did not change the effect estimates (supplementary figure S2). Additional adjustments for lifestyle factors and self-reported mental health did not substantially change the effect sizes (supplementary figure S3). We did not observe significant differences across age groups, sex and educational levels (supplementary figure S4).

In a supplementary analysis, before mutual adjustment of the individual resources, some associations were observed for procedural justice, leadership quality and co-worker support (supplementary figure S5A). Most of these associations attenuated after mutual adjustment: only intermediately high level of procedural justice (HR 0.84, 95% CI 0.74–0.96) and high level of support from colleagues (HR 0.87, 95% CI 0.78–0.97) remained associated with a lower risk of CVD after mutual adjustment (supplementary figure S5B).

## Discussion

This analysis of individual-level data on almost 140 000 persons from three Nordic cohort studies identified a consistent pattern including five classes of workplace psychosocial resource across follow-up waves, employment sectors and countries. About 13% of the employees experienced low levels of all studied workplace resources, suggesting a potential for improvement. Our findings show a consistent protective effect of workplace resources on overall CVD, most prominently for myocardial infarction and CBD.

A moderate effect on myocardial infarction was observed when intermediate level of vertical resources (ie, procedural justice and leadership quality) were combined with high level of horizontal resources (ie, culture of collaboration and co-worker support), in line with previous research on specific aspects of workplace resources and hospitalization/death due to myocardial infarction (7, 19, 20). Lack of social support in general may also be associated with cardiac mortality or all-cause mortality (37). Our findings add to the evidence by comprehensively identifying clustering patterns of resources across several key vertical and horizontal psychosocial resources.

We found a lower risk of developing CBD when perceiving high level of horizontal resources (ie, culture of collaboration and co-worker support). To the best of our knowledge, we are the first longitudinal study demonstrating an association between these workplace resources and risk of CBD. André-Petersson et al (21) found no association between workplace social support and CBD, but based on only 58 cases, much less than 1097 cases in our study. Our findings need to be replicated in other studies.

We found a stronger effect for CBD than CHD, similar to the one shown in a previous multicohort study concerning long working hours (38), while the CI between CHD and CBD were highly overlapping. These issues require careful investigations in future studies.

The underlying mechanisms for the potentially protective vascular effects remain to be uncovered. Earlier research have shown a favorable level of workplace social support (16) and relational justice (17) in connection with a lower ambulatory blood pressure. Low workplace social support has been found to be associated with a higher level of interleukin-6 (13). Other possible mechanistic pathways include indirect effect via health-related behaviors (10, 39–41) and mental health problems (42).

### Public health implications

It is interesting that there was no evidence of independent associations between each individual resource and CVD in mutually-adjusted models. When clustering was considered, the associations with CVD became clear and similar to those previously found for type-2 diabetes (14). This emphasizes the importance of exploring clustering of resources instead of singling out individual effects and is consistent with findings from a systematic review, which highlighted the importance of multi-level workplace interventions (23), ie, to intervene on vertical and horizontal dimensions of resources at the same time.

Considering the average annual incidence of 26.8 per 10 000 persons, the relative differences, such as a 16% lower risk of developing incident CVD and the absolute differences, such as 3.4 lower incidence per 10 000 person-years when comparing the ‘intermediate vertical+high horizontal’ with the ‘general low’, should be interpreted cautiously. The public health importance, if causal, will depend on the distribution of resource classes across settings. For example, compared with public sector employees (ie, FPS), the general working population (ie, WEHD and SLOSH) contained a smaller proportion of workers in the ‘general low’ resource class and fewer women. Public and private sector employees may differ in their perception of job, communication formalization and objective-oriented results (43). Male and female employees may experience different employment and working conditions (44) and have diverged perception on some resources (16, 45). A deeper understanding of the distribution of resource clusters in specific settings is needed to develop targeted work-related CVD preventions in different types of workplaces, eg, among private and public sector employees.

Interestingly, the lowest risk of CVD was often found in the “intermediate vertical+high horizontal” resource group rather the highest resource group. This suggests that not all resources at work are equally important in reducing the risk of CVD. Some studies suggest that workplace social support may in some cases be unhelpful and even trigger stress (46). However, the CI between “general high” and “intermediate vertical+high horizontal” resource groups were overlapping and thus not statistically different. More research is needed to understand whether pursuing the highest level of resources at work is always beneficial for employee health.

### Theoretical relevance

We selected workplace psychosocial resources at group (eg, team climate), leader (eg, leadership quality) and organizational levels (eg, perceived organizational support), following a recently proposed theoretical framework for workplace resources by Nielsen et al (23). This review also showed that these workplace resources may be associated with better employees’ job performance and well-being (23). Consistent with existing evidence of psychosocial resources at work (23), we identified clustering of the four resources according to vertical and horizontal dimensions. Future research may be required to disentangle the potential interactions among these resources to facilitate the design of cost-effective multilevel interventions.

### Limitations and strengths

Some limitations merit careful consideration. Workplace psychosocial resources were measured by self-assessment at baseline and job changes were not accounted for. Although omitting the effect of time-varying resources and time-varying confounders may have resulted in an underestimation of the association, when we restricted the follow-up lengths to the first four years, the risk estimates remained similar, suggesting this to be of limited concern. FPS contained information only on in-patient visits and thus more likely to capture severe CVD cases, potentially contributing to an underestimation of the effect (47). While the same questionnaires were used in SLOSH and FPS, WEHD used slightly different instruments to measure culture of collaboration, procedural justice and leadership quality. This is an unlikely source of major bias because the three cohorts showed very similar patterns of resource clusters and associations with CVD. Although the point estimates were higher in SLOSH and WEHD than in FPS, no statistically significant heterogeneity was detected in cohort-specific effect estimates. We estimated four common group-, leader- and organization-level workplace psychosocial resources using a data-driven approach to detect clustering of workplace resources. This approach may be sensitive to the categorization and selection of the resource items. Future research is therefore needed to test the robustness of the clusters when using different categorizations. Some more detailed aspects of resources, such as perceiving or receiving co-worker’s support was not considered, and may be considered as a limitation (37). While resources tend to highly intercorrelate, a more comprehensive mapping of resources may be needed in the future.

The strengths of our study include the large sample size with long follow-up, which allowed us to perform analyses on specific subtypes of CVD as well as conduct a range of sensitivity analyses with sufficient statistical power. We were able to perform a nearly complete follow-up by linking survey data to nationwide registries to identify new cases of definite CHD and CBD. The inclusion of participants from both the general working population and public sector employees of three Scandinavian countries further provided a sufficient number of participants from both sexes and ensured the diversity of industry, job type and employment sectors, which assures the generalizability of our findings to similar contexts.

### Concluding remarks

Our study identified five distinct workplace psychosocial resource clusters with different levels of resources, consistent across countries and employment sectors. Employees with favorable workplace psychosocial resources, especially intermediate and high vertical and high horizontal resources, were at a lower risk of CVD. Further research is needed to determine whether interventions to improve workplace psychosocial resources could be beneficial to vulnerable groups with established CVD risk factors.

### Funding

The Danish Working Environment Foundation supported this study (grant 13-2015-09). TX was supported by a grant from the Swedish Research Council for Health, Working Life and Welfare (grant 2020-00040). MK was supported by research grant from NordForsk (grant 70521, the Nordic Research Programme on Health and Welfare), the UK Medical Research Council (grant MRC S011676), the US National Institute on Aging (NIA) (grant R01AG056477), the Academy of Finland (grant 329202), and Finnish Work Environment Fund (grant 190424). LMH was supported by a grant from the Swedish Research Council for Health, Working Life and Welfare (grant 2019-01318). MV was supported by Academy of Finland (grant 329201). JV was supported by the Academy of Finland (grant 321409 and 329240). JE was supported by the Academy of Finland (grant 329200), the Finnish Work Environment Fund (grant 200097), Government’s Analysis, Assessment and Research Activities (grant VN/14606/2019), and the Academy of Finland Strategic Research Council (grant 336004).

### Disclosures

AJC is an employee at Novo Nordisk A/S and moved to Novo Nordisk at the end of the project. Her current employer has no role in the study design, analyses and results interpretation. No other potential conflicts of interest relevant to this article were declared.

## Supplementary material

Supplementary material
