# Supplementary material for: Workplace psychosocial resources and risk of cardiovascular disease among employees: a multi-cohort study of 135 669 participants
Source: Scand J Work Environ Health. 2022 Oct 29;48(8):621–31. doi: 10.5271/sjweh.4042 (PMC10546613; doi:10.5271/sjweh.4042)
Supplement: Supplementary material [file SJWEH-48-621-S001.pdf]

Workplace psychosocial resources and risk of cardiovascular disease among employees: a multi-cohort study of 136 831 participants[1]

by Tianwei Xu, PhD,[2] Reiner Rugulies, PhD, Jussi Vahtera, PhD, Jaana Pentti, MSc, Jimmi Mathisen, MSc, Theis Lange, PhD, Alice J Clark, PhD, Linda L Magnusson Hanson, PhD, Hugo Westerlund, PhD, Jenni Ervasti, PhD, Marianna Virtanen, PhD, Mika Kivimäki, PhD, Naja H Rod, PhD

1. Supplementary material
2. Correspondence: Tianwei Xu, Stress Research Institute, Stockholm University, 106 91 Stockholm, Sweden. [E-mail: tianwei.xu@psychology.su.se]

Table of Contents

Supplementary Text S1. The choice of measurements, dimensions and cut-offs of workplace psychosocial resources....2

Supplementary Text S2. Measurement of subtypes of cardiovascular disease....3

Supplementary Text S3. Measurement of covariates....4

Supplementary Table S1. Items measuring workplace resources....5

Supplementary Table S2. Correlations and agreements between a single-item measurement and the using the full scale....6

Supplementary Figure S1. Comparison of latent class models....7

Supplementary Figure S2. Difference in follow-up lengths....8

Supplementary Figure S3. Additional adjustment for lifestyle factors and prior mental disorders (n=100,517, number of cvd events=1098)....9

Supplementary Figure S4. Fixed effect model (n=135,669) on the association between clustering of workplace social resources and incident cvd, stratified by age group, sex and educational level....10

Supplementary Figure S5. Association between each individual workplace psychosocial resource and incident cvd, before (a) and after (b) mutually adjusting for each other, after adjustment for age, sex, country of birth, educational level, marital status, pre-existing comorbidity, pre-existing mental disorders and types of employment contract. fixed effect meta-analysis....11

Appendix. Statistical syntax for analysis....12

SAS CODES....12

R CODES....14

Supplementary Text S1. The choice of measurements, dimensions and cut-offs of workplace psychosocial resources.

Similar item for ‘support from colleagues’ (e.g. “my colleagues are there for me”) has been used in multicohort analyses on type 2 diabetes, including cohort studies from Sweden, United Kingdom and France (1). We applied the same cut-off for high versus low support from colleagues as in these studies, classifying e.g. response options ‘completely agree’ and ‘somewhat agree’ as high support. The measure of ‘collaboration’ is part of the measurement of workplace social capital (2), in which self-reported workplace social capital was associated with a lower risk of chronic hypertension among men and mortality among men and women in previous studies (3, 4). We used median cut-offs for high versus low levels of collaboration as in studies on workplace social capital. In previous studies, the measure of ‘procedural justice’ (Moorman scale) has been divided into four (5) or three (6) categories while analyses of leadership quality have typically been based on quartiles (5, 7, 8). We used quartiles for both constructs. The measurement of ‘leadership’ included four dimensions in SLOSH and FPS in agreement with a larger IPD-Work multicohort research program, in which the current study is participating, and a previous large-scale multicohort study (7). In WEHD, the dimensions of leadership quality were slightly different due to data constrains, but this operationalization has also been used previously (8).

References

1. Mortensen J, Clark AJ, Lange T, Andersen GS, Goldberg M, Ramlau-Hansen CH, et al. Informal caregiving as a risk factor for type 2 diabetes in individuals with favourable and unfavourable psychosocial work environments: A longitudinal multi-cohort study. Diabetes & Metabolism. 2018;44(1):38-44.
2. Kouvonen A, Kivimäki M, Vahtera J, Oksanen T, Elovainio M, Cox T, et al. Psychometric evaluation of a short measure of social capital at work. BMC public health. 2006;6(1):251.
3. Oksanen T, Kawachi I, Jokela M, Kouvonen A, Suzuki E, Takao S, et al. Workplace social capital and risk of chronic and severe hypertension: a cohort study. Journal of hypertension. 2012;30(6):1129-36.
4. Oksanen T, Kivimäki M, Kawachi I, Subramanian S, Takao S, Suzuki E, et al. Workplace social capital and all-cause mortality: A prospective cohort study of 28 043 public-sector employees in Finland. American journal of public health. 2011;101(9):1742-8.
5. Persson V, Eib C, Bernhard-Oettel C, Leineweber C. Effects of procedural justice on prospective antidepressant medication prescription: a longitudinal study on Swedish workers. BMC public health. 2020;20:1-9.
6. Lallukka T, Halonen JI, Sivertsen B, Pentti J, Stenholm S, Virtanen M, et al. Change in organizational justice as a predictor of insomnia symptoms: longitudinal study analysing observational data as a non-randomized pseudo-trial. International journal of epidemiology. 2017;46(4):1277-84.
7. Madsen IEH, Hanson LLM, Rugulies R, Theorell T, Burr H, Diderichsen F, et al. Does good leadership buffer effects of high emotional demands at work on risk of antidepressant treatment? A prospective study from two Nordic countries. Social psychiatry and psychiatric epidemiology. 2014;49(8):1209-18.
8. Sørensen JK, Framke E, Clausen T, Garde AH, Johnsen NF, Kristiansen J, et al. Leadership Quality and Risk of Long-term Sickness Absence Among 53,157 Employees of the Danish Workforce. Journal of occupational and environmental medicine / American College of Occupational and Environmental Medicine. 2020;62(8):557-65.

Supplementary Text S2. Measurement of subtypes of cardiovascular disease

Myocardial infarction:

Hospital registers: using main diagnosis of ICD-10 I21-22, or 410 in ICD-9 or ICD8 41.

Death register: using any main cause of death in acute coronary death of ICD-10 I20-25, or 410–414 in ICD-9, or ICD 8 410-414.

Ischaemic stroke:

Hospital registers and death register: using main diagnosis of ICD-10 I63 or ICD-9 433-434, or ICD8 433-434.

Haemorrhagic stroke:

Hospital registers and death register: using main diagnosis of codes ICD-10 I61, I62 or ICD-9 431 or ICD8 431.

Supplementary Text S3. Measurement of covariates

Information on key covariates were extracted from the national register in Denmark, Finland and Sweden, except that marital status in Finland and employment contract in Sweden were measured by self-report. Pre-existing comorbidities according to Charlson Comorbidity Index (table below) and mental disorders were detected using ICD codes from national patient register.

ICD10 for diagnosed mental disorders: F01-F99.

ICD codes for Charlson Comorbidity Index, calculated using both the primary and secondary diagnosis where applicable.				
Condition	Weights	ICD-8	ICD-9	ICD-10
Acute myocardial infarction	1	410	410, 412	I21, I22, I252
Congestive heart failure	1	427.09; 427.10; 427.11; 427.19; 428.99; 782.49	428	I50
Peripheral vascular disease	1	440; 441; 442; 443; 444; 445	441, 4439, 7854, V434	I71, I790, I739, R02, Z958, Z959
Cerebral vascular accident	1	430–438	430–438	I60, I61, I62, I63, I65, I66, G450, G451, G452, G458, G459, G46, I64, G454, I670, I671, I672, I674, I675, I676, I677, I678, I679, I681, I682, I688, I69
Dementia	1	290.09–290.19; 293.09	290	F00, F01, F02, F051
Pulmonary disease	1	490–493; 515–518	490, 491, 492, 493, 494, 495, 496, 500, 501, 502, 503, 504, 505	J40, J41, J42, J44, J43, J45, J46, J47, J67, J44, J60, J61, J62, J63, J66, J64, J65
Connective tissue disorder	1	712; 716; 734; 446; 135.99	7100, 7101, 7104, 7140, 7141, 7142, 71481(now 5171), 725	M32, M34, M332, M053, M058, M059, M060, M063, M069, M050, M052, M051, M353
Peptic ulcer	1	530.91; 530.98; 531–534	531, 532, 533, 534	K25, K26, K27, K28
Liver disease	1	571; 573.01; 573.04	5712, 5714, 5715, 5716	K702, K703, K73, K717, K740, K742, K746, K743, K744, K745
Diabetes	1	249.00; 249.06; 249.07; 249.09 250.00; 250.06; 250.07; 250.09	2500,2501, 2502, 2503, 2507	E109, E119, E139, E149, E101, E111, E131, E141, E105, E115, E135, E145
Diabetes complications	2	249.01–249.05; 249.08 250.01–250.05; 250.08	2504, 2505, 2506	E102, E112, E132, E142 E103, E113, E133, E143 E104, E114, E134, E144
Paraplegia	2	344	342, 3441	G81 G041, G820, G821, G822
Renal disease	2	403; 404; 580–583; 584; 590.09; 593.19; 753.10–753.19; 792	582, 5830, 5831, 5832, 5833, 5835, 5836, 5837, 5834, 585, 586, 588	N03, N052, N053, N054, N055, N056, N072, N073, N074, N01, N18, N19, N25
Cancer	2	140–194<br>204–207<br>200–203; 275.59	14, 15, 16, 18, 170, 171, 172, 174, 175, 176, 179, 190, 191, 192, 193, 194, 1950, 1951, 1952, 1953, 1954, 1955, 1958, 200, 201, 202, 203, 204, 205, 206, 207, 208	C0, C1, C2, C3, C40, C41, C43, C45, C46, C47, C48, C49, C5, C6, C70, C71, C72, C73, C74, C75, C76, C80, C81, C82, C83, C84, C85, C883, C887, C889, C900, C901, C91, C92, C93, C940, C941, C942, C943, C9451, C947, C95, C96
Metastatic cancer	6	195–198; 199	196, 197, 198, 1990, 1991	C77, C78, C79, C80
Severe liver disease	3	070.00; 070.02; 070.04; 070.06; 070.08; 573.00; 456.00–456.09	5722, 5723, 5724, 5728	K729, K766, K767, K721
HIV	6	79.83	042, 043, 044	B20, B21, B22, B23, B24

Body mass index: calculated based on self-reported weight and height.
Self-reported mental health: Symptom Checklist Core depression-6 in SLOSH, Major Depression Inventory in WEHD, General Health Questionnaire in FPS.
Smoking: self-reported current smoking status.
Risky lcohol Consumption: Women/men drinking exceeding 14/21 alcohol units (12 g of alcohol per unit) per week meant excessive alcohol consumption.
Physical inactivity: Physical inactivity was observed if participants reported if having <2.0 metabolic equivalent task hours per day (corresponding to approximately 30 minutes of walking) in WEHD and FPS or reported physical inactivitiy in SLOSH.

Supplementary Table S1. Items measuring workplace resources.

Type of resource	WEHD	FPS	SLOSH
**Support from colleagues**	How often do you and your colleagues collaborate when you are facing problems that require a solution?<br>(5 point likert scale)<br>'All the time' or 'often',<br>Vs. 'sometimes', 'rarely' or 'never'	How much do you think your teammates support and help you?<br>(5 point likert scale)<br>'Very much', 'a lot',<br>Vs. 'neither much nor a little', 'a little', 'little'	My colleagues are there for me.<br>(4 point likert scale)<br>'Totally agree', or 'somewhat agree',<br>Vs. 'somewhat disagree', 'totally disagree'
**Culture of collaboration**	How often do you and your colleagues help each other for achieving the best possible results?<br>(5 point likert scale)<br>'All the time' or 'often',<br>Vs. 'sometimes', 'rarely' or 'never'	1. Do members of the work unit build on each other's ideas in order to achieve the best possible outcome?<br>2. People in the work unit cooperate in order to help develop and apply new ideas.<br>(Cronbach's alpha=0.77)<br>(5 point likert scale)<br>'Very much', 'a lot', 'neither much nor a little', 'a little', 'little'<br>Mean separation<br>Missing: if 2 items were missing	1. Members of the work unit build on each other's ideas in order to achieve the best possible outcome.<br>2. People in the work unit cooperate in order to help develop and apply new ideas.<br>(Cronbach's alpha=0.87-0.88)<br>(4 point likert scale)<br>'Totally agree', or 'somewhat agree', 'somewhat disagree', 'totally disagree'<br>Mean separation<br>Missing: if 2 items were missing
**Leadership quality**	How often:<br>1. does your immediate manager explain the company's objectives, so you understand what they mean for your work tasks?<br>2. do you have sufficient authority in relation to your responsibilities at work?<br>3. does your immediate manager take the time to engage in your professional development?<br>4. does your immediate manager involve you in the planning of your work?<br>5. does your immediate manager give you the necessary feedback (praise and criticism) for your work?<br>6. is your work recognized and appreciated by the management?<br>7. do you get the necessary help and support from your immediate manager?<br>8. can you trust the information that comes from the management?<br>(Cronbach's alpha=0.89)<br>(5 point likert scale)<br>'All the time', 'often', 'sometimes', 'rarely', 'never'<br>Mean separation<br>Missing: if ≥ 4 items were missing	1. Our superior does not care about the feelings of the employees.<br>2. Our superior listens to his subordinates' opinions in important cases.<br>3. Our superior rewards good work effort.<br>4. Our superior informs us in good time on decisions taken and their consequences.<br>(Cronbach's alpha=0.88)<br>(5 point likert scale)<br>'Totally agree', 'somewhat agree', 'not agree nor disagree', 'somewhat disagree', 'totally disagree'<br>Mean separation<br>Missing: if ≥ 2 items were missing	1. My boss shows that he/she cares how things are for me and how I feel.<br>2. Your manager genuinely listen to what you have to say.<br>3. I get the acknowledgement I deserve from my superiors.<br>4. My boss gives me the information I need.<br>(Cronbach's alpha=0.84)<br>(4 point likert scale)<br>'Totally agree', 'somewhat agree', 'somewhat disagree', 'totally disagree'<br>Mean seperation<br>Missing: if ≥ 2 items were missing
**Procedural Justice**	How often the concerns of all those affected by the decision were heard.<br>(5 point likert scale)<br>'All the time'<br>Vs. 'often', or 'sometimes',<br>Vs. 'rarely',<br>Vs. 'never'	1. Procedures are designed to collect accurate information necessary for making decisions.<br>2. Procedures are designed to provide opportunities to appeal or challenge the decision.<br>3. Procedures are designed to hear the concerns of all those affected by the decision.<br>4. Procedures are designed to generate standards so that decisions can be made with consistency.<br>(Cronbach's alpha=0.90)<br>(5 point likert scale)<br>'Totally agree', 'somewhat agree', 'not agree nor disagree', 'somewhat disagree', 'totally disagree'<br>Mean separation<br>Missing: if ≥ 2 items were missing	1. Decisions are taken on the basis of correct information.<br>2. Bad decisions can be revoked or changed.<br>3. All sides affected by the decision are represented.<br>4. Decisions taken are consistent (the same rules apply to everyone).<br>5. Everyone is entitled to give their opinion in matters of immediate personal concern.<br>6. Feedback is provided regarding the consequences of decisions and people are informed accordingly.<br>7. It is possible to obtain a more detailed account of the information that underlies decisions, if needed.<br>(Cronbach's alpha=0.91)<br>(5 point likert scale)<br>'Totally agree', 'somewhat agree', 'neither agree nor disagree', 'somewhat disagree', 'totally disagree'<br>Mean seperation<br>Missing: if ≥ 4 items were missing

Supplementary Table S2. Correlations and agreements between a single-item measurement and the using the full scale.

Variables	Item in WEHD	Item selected in FPS	Item selected in SLOSH	Spearman correlation coefficients*	Cohen's κ **
Procedural justice	How often the concerns of all those affected by the decision were heard.	Procedures are designed to hear the concerns of all those affected by the decision.	All sides affected by the decision are represented.	FPS: 0.86<br>SLOSH: 0.80	FPS: 0.54<br>SLOSH: 0.49
Culture of collaboration	How often do you and your colleagues help each other for achieving the best possible results?	Do members of the work unit build on each other's ideas in order to achieve the best possible outcome?	Members of the work unit build on each other's ideas in order to achieve the best possible outcome.	FPS: 0.89<br>SLOSH: 0.93	FPS: 0.69<br>SLOSH: 0.89

* Correlation between the single item and the sum of the scale.

** Cohen's κ calculated by treating the variables into two (culture of collaboration) or four (procedural justice) categories, as practiced in the current study; Weighted kappa was calculated for procedural justice to account for the ordering nature of the variable.

Abbreviations: WEHD=Work Environment and Health in Denmark study; FPS=Finnish Public Sector study; SLOSH=Swedish Longitudinal Occupational Survey of Health.

Supplementary Figure S1. Comparison of latent class models.

Cohort	BIC for 2 classes	BIC for 3 classes	BIC for 4 classes	BIC for 5 classes	BIC for 6 classes
WEHD	337100.2	330751.1	328800.2	328165.6	Not convergent
FPS	486247.4	483256.1	480956.2	480704.7	Not convergent
SLOSH	100805.2	100076.2	99970.9	99970.5	Not convergent
Abbreviation: BIC= Bayesian Information Criterion, the lower the better.					

BIC decreased from 2-class model to 5-class model and when adding a sixth class, models in WEHD and FPS did not converge and BIC for 6 classes in SLOSH was larger than that for 5 classes. Considering the comparability across cohorts and the interpretability of the classes, we only compared between the four- and five-class solutions.

Four class solution:

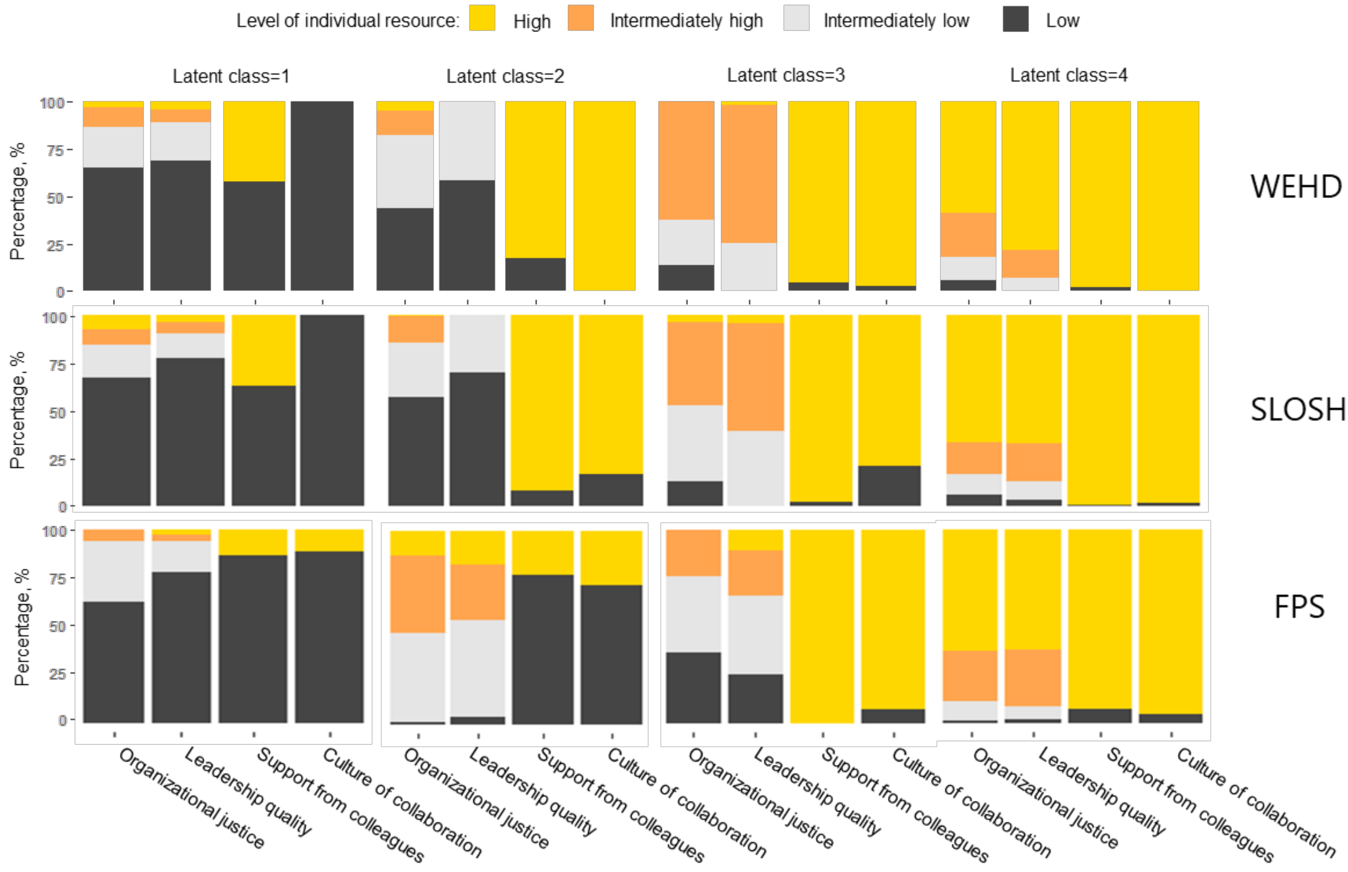

WEHD and SLOSH already showed similar patterns in the four-class solution. The only difference across the three cohorts in the four-class solution concerns the latent class 2. In FPS, we identified “intermediate vertical+low horizontal” class, and in SLOSH and WEHD, we identified the “low vertical+high horizontal” class.

Supplementary Figure S2. Difference in follow-up lengths.

	Total (n)	Events (n)	HR (95% CI)
Using the first four years of follow−up			
General low	18040	140	Reference
Intermediate vertical + low horizontal	14394	96	0.94 (0.72 − 1.22)
Low vertical + high horizontal	22441	148	0.93 (0.72 − 1.19)
Intermediate vertical + high horizontal	42900	277	0.88 (0.71 − 1.09)
General high	37894	239	0.92 (0.74 − 1.13)
Excluding cases developed during the first year			
General low	15478	414	Reference
Intermediate vertical + low horizontal	12638	292	0.91 (0.78 − 1.06)
Low vertical + high horizontal	16623	233	0.89 (0.77 − 1.03)
Intermediate vertical + high horizontal	32708	479	0.84 (0.73 − 0.97)
General high	30074	523	0.89 (0.78 − 1.01)

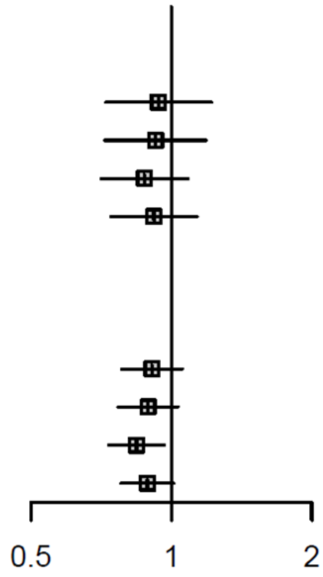

Supplementary Figure S3. Additional adjustment for lifestyle factors and prior mental disorders (N=100,517, Number of CVD events=1098).

	Total(n)	Events(n)	HR (95% CI)
Main adjustment			
General low	14010	208	Reference
Intermediate vertical + low horizontal	10597	155	0.97 (0.79 – 1.21)
Low vertical + high horizontal	15867	161	1.05 (0.86 – 1.29)
Intermediate vertical + high horizontal	30350	278	0.89 (0.74 – 1.06)
General high	29693	296	0.86 (0.73 – 1.03)
Main adjustment + BMI			
General low	14010	208	Reference
Intermediate vertical + low horizontal	10597	155	0.97 (0.79 – 1.19)
Low vertical + high horizontal	15867	161	1.05 (0.86 – 1.29)
Intermediate vertical + high horizontal	30350	278	0.90 (0.75 – 1.07)
General high	29693	296	0.87 (0.73 – 1.04)
Main adjustment + physical activity			
General low	14010	208	Reference
Intermediate vertical + low horizontal	10597	155	0.96 (0.78 – 1.18)
Low vertical + high horizontal	15867	161	1.06 (0.86 – 1.29)
Intermediate vertical + high horizontal	30350	278	0.89 (0.75 – 1.07)
General high	29693	296	0.87 (0.73 – 1.04)
Main adjustment + alcohol consumption			
General low	14010	208	Reference
Intermediate vertical + low horizontal	10597	155	0.96 (0.78 – 1.18)
Low vertical + high horizontal	15867	161	1.05 (0.86 – 1.29)
Intermediate vertical + high horizontal	30350	278	0.89 (0.74 – 1.06)
General high	29693	296	0.86 (0.72 – 1.03)
Main adjustment + smoking			
General low	14010	208	Reference
Intermediate vertical + low horizontal	10597	155	0.97 (0.79 – 1.20)
Low vertical + high horizontal	15867	161	1.05 (0.86 – 1.29)
Intermediate vertical + high horizontal	30350	278	0.90 (0.76 – 1.08)
General high	29693	296	0.87 (0.73 – 1.04)
Main adjustment + mental disorders			
General low	14010	208	Reference
Intermediate vertical + low horizontal	10597	155	0.98 (0.80 – 1.21)
Low vertical + high horizontal	15867	161	1.08 (0.88 – 1.32)
Intermediate vertical + high horizontal	30350	278	0.92 (0.77 – 1.11)
General high	29693	296	0.91 (0.76 – 1.09)
Main adjustment + all variables			
General low	14010	208	Reference
Intermediate vertical + low horizontal	10597	155	0.99 (0.81 – 1.22)
Low vertical + high horizontal	15867	161	1.08 (0.88 – 1.32)
Intermediate vertical + high horizontal	30350	278	0.94 (0.79 – 1.13)
General high	29693	296	0.92 (0.77 – 1.10)

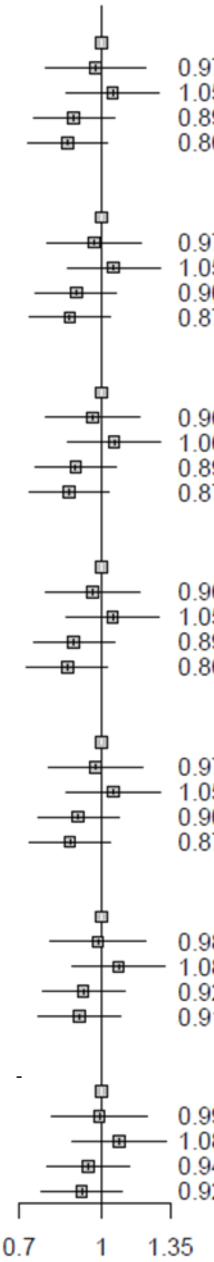

Note 1: Main adjustment = age, sex, educational level, country of birth, marital status, pre-existing comorbidity score, pre-existing mental disorders and types of employment contract.

Note 2: Information on lifestyle factors and self-reported mental health was not available in FPS 2000, 2006 and 2010. The absence of FPS wave 2000, 2006 and 2010 in this analysis for adjustments also lead to a fewer number of CVD events than that in the main analysis (CVD events=2191).

Note 3: All analyses were performed based on the same sample size to allow a better comparison across adjustments.

Supplementary Figure S4. Fixed effect model (N=135,669) on the association between clustering of workplace social resources and incident CVD, stratified by age group, sex and educational level.

	Total(n)	Events(n)	HR (95% CI)	IRD (95% CI)
**55 or younger**				
General low	14813	311	Reference	Reference
Intermediate vertical + low horizontal	11988	229	0.96 (0.82 – 1.12)	−0.8 (−4.3 – 2.6)
Low vertical + high horizontal	17418	160	0.94 (0.80 – 1.10)	−1.0 (−4.7 – 2.6)
Intermediate vertical + high horizontal	33393	320	0.84 (0.74 – 0.96)	−2.8 (−5.7 – 0.2)
General high	30144	390	0.95 (0.83 – 1.08)	−0.6 (−3.5 – 2.4)
**Older than 55**				
General low	3227	141	Reference	Reference
Intermediate vertical + low horizontal	2406	88	0.87 (0.72 – 1.06)	−15.8 (−30.9 – −0.8)
Low vertical + high horizontal	5023	114	0.88 (0.74 – 1.05)	−11.2 (−28.3 – 6.0)
Intermediate vertical + high horizontal	9507	238	0.88 (0.75 – 1.02)	−9.5 (−23.8 – 4.8)
General high	7750	199	0.82 (0.70 – 0.96)	−15.6 (−29.1 – −2.0)
**Men**				
General low	6053	213	Reference	Reference
Intermediate vertical + low horizontal	4181	132	0.90 (0.75 – 1.07)	−6.6 (−14.6 – 1.5)
Low vertical + high horizontal	8749	154	0.95 (0.81 – 1.10)	−2.4 (−12.7 – 7.9)
Intermediate vertical + high horizontal	15878	290	0.90 (0.79 – 1.03)	−2.0 (−9.6 – 5.6)
General high	12010	249	0.90 (0.78 – 1.03)	−2.7 (−10.3 – 4.8)
**Women**				
General low	11987	239	Reference	Reference
Intermediate vertical + low horizontal	10213	185	0.96 (0.81 – 1.14)	−2.0 (−5.8 – 1.9)
Low vertical + high horizontal	13692	120	0.84 (0.69 – 1.03)	−3.1 (−7.0 – 0.9)
Intermediate vertical + high horizontal	27022	268	0.80 (0.68 – 0.93)	−4.0 (−7.4 – −0.6)
General high	25884	340	0.87 (0.75 – 1.01)	−2.3 (−5.7 – 1.0)
**Low**				
General low	2317	101	Reference	Reference
Intermediate vertical + low horizontal	1769	69	0.87 (0.66 – 1.15)	−8.9 (−22.3 – 4.6)
Low vertical + high horizontal	2360	52	0.95 (0.73 – 1.22)	−2.1 (−20.0 – 15.8)
Intermediate vertical + high horizontal	4510	114	0.89 (0.72 – 1.10)	−6.6 (−20.3 – 7.1)
General high	4548	121	0.90 (0.73 – 1.11)	−6.8 (−19.2 – 5.7)
**Medium**				
General low	7423	182	Reference	Reference
Intermediate vertical + low horizontal	5670	136	1.04 (0.87 – 1.25)	0.4 (−5.9 – 6.7)
Low vertical + high horizontal	9473	115	0.91 (0.76 – 1.10)	−4.5 (−10.9 – 2.0)
Intermediate vertical + high horizontal	17533	243	0.91 (0.78 – 1.06)	−3.2 (−8.5 – 2.1)
General high	17708	232	0.94 (0.80 – 1.10)	−1.3 (−6.6 – 4.0)
**High**				
General low	8300	169	Reference	Reference
Intermediate vertical + low horizontal	6955	112	0.84 (0.68 – 1.03)	−3.8 (−8.1 – 0.4)
Low vertical + high horizontal	10608	107	0.88 (0.73 – 1.07)	−0.8 (−5.5 – 3.9)
Intermediate vertical + high horizontal	20857	201	0.78 (0.66 – 0.92)	−2.7 (−6.7 – 1.2)
General high	18368	236	0.83 (0.71 – 0.98)	−2.1 (−5.8 – 1.7)

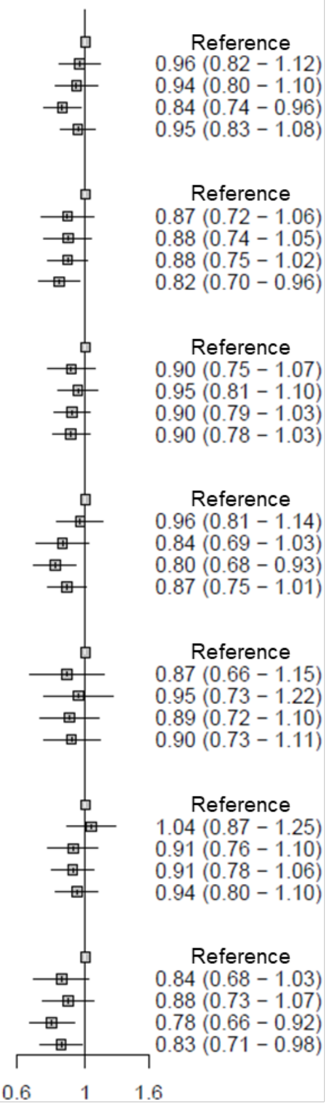

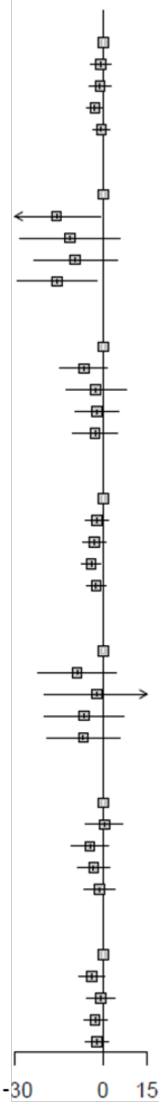

Supplementary Figure S5. Association between each individual workplace psychosocial resource and incident CVD, before (A) and after (B) mutually adjusting for each other, after adjustment for age, sex, country of birth, educational level, marital status, pre-existing comorbidity, pre-existing mental disorders and types of employment contract. Fixed effect meta-analysis.

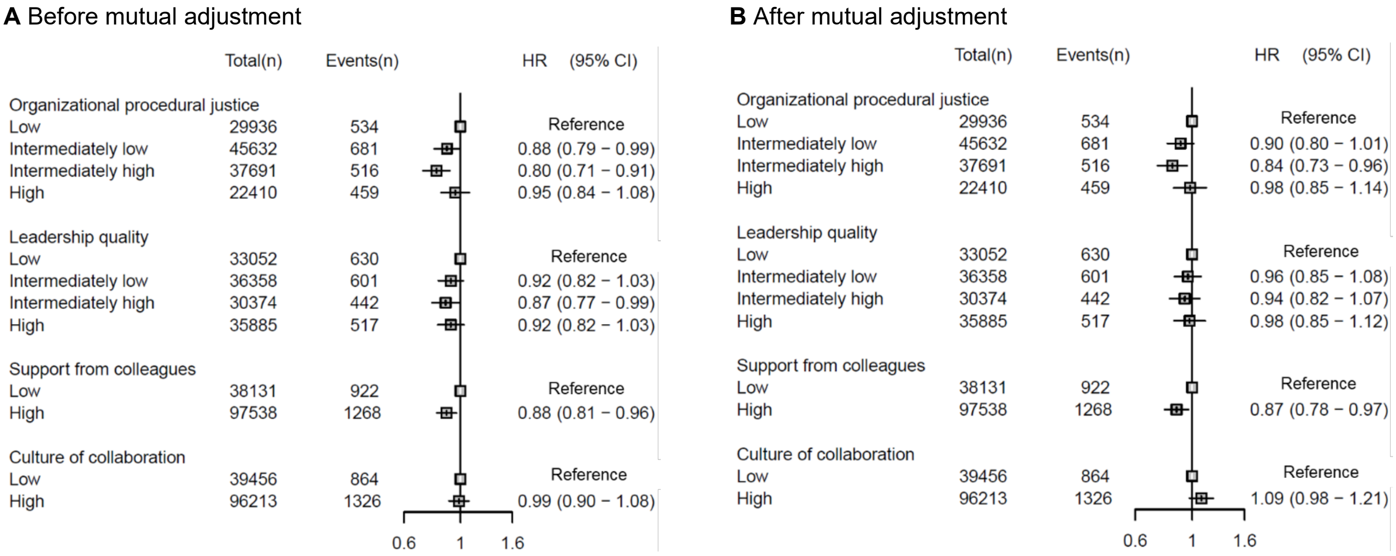

Appendix. Statistical syntax for analysis

SAS codes

```
libname result2 'H:\results4';

data &disease.1;
set &disease.1;
if ageb>= 55 then agegrp=1;
if 18<=ageb<55 then agegrp=0;
rename &disease.event=&disease;
run;


data &disease._rev;
set &disease.1;
ageb=ageb+1;
if ageb>=age&disease. then delete;
rename &disease.event=&disease;
run;

data &disease._4y;
set &disease.1;
&disease.time=age&disease.-ageb;
if &disease.=1 and &disease.time>4 then do; &disease.time=4; &disease.=0; end;
if &disease.=0 and &disease.time>4 then do; &disease.time=4; &disease.=0; end;

age&disease.=ageb+&disease.time;
rename &disease.event=&disease;

run;

%macro main(data,dis,r);
proc phreg data=&data fast ;
ods exclude none;
title "&r";
class &r(ref='1') wave(ref='4') mental(ref='0') sex(ref='2')  edu(ref='1') marital(ref='G') employment(ref='permanent') rankorg(ref='1')
rankleader(ref='1') support(ref='1') co(ref='1');
%if &r = resource %then %do;
model (ageb,age&disease.)*&disease.(0)= &r wave sex marital edu score employment mental/rl ; %end;
%if &r =rankorg or &r =rankleader or &r =support or &r =co %then %do;
model (ageb,age&disease.)*&disease.(0)= &r wave sex marital edu score employment rankorg rankleader support co mental/rl ; %end;
ods output  ParameterEstimates=parameterestimates;
run;

proc print data= ParameterEstimates; run;

%mend;
%main(&disease.1,&disease.,resource)
%main(&disease.1,&disease.,rankorg)
%main(&disease.1,&disease.,rankleader)
%main(&disease.1,&disease.,co)
%main(&disease.1,&disease.,support)
%main(&disease._4y,&disease.,resource)
ods exclude none;


%main(&disease._rev,&disease.,resource)
%main(&disease._rev,&disease.,rankorg)
%main(&disease._rev,&disease.,rankleader)
%main(&disease._rev,&disease.,co)
%main(&disease._rev,&disease.,support)


%macro interaction1(resource,v);
proc phreg data=&data fast ;
ods exclude all;
class &resource(ref='1') wave(ref='4') mental(ref='0') sex(ref='2')  edu(ref='1')  marital(ref='G') employment(ref='permanent') agegrp(ref='0');
model (ageb,age&disease.)*&disease.(0)= &resource wave mental agegrp &resource*&v  sex marital edu employment score/rl ;
hazardratio &resource /at (&v=all) diff=ref;
```

```
ods output HazardRatios=HR  ModelANOVA=pvalue;
run;

proc print data= HazardRatios; run;

%mend;
%let data=&disease.1;
%interaction1(resource,agegrp);
%interaction1(resource,sex);
%interaction1(resource,edu);


ods exclude none;

%macro adjustment(disease,r,v);
proc phreg data=&data fast ;
ods exclude none;
title "Adjustment: &v";
class &r(ref='1') wave(ref='4') sex(ref='2') mental(ref='0') edu(ref='1')  marital(ref='G') employment(ref='permanent')  phy(ref='0') smoke(ref='0')
alcohol(ref='0');
model (ageb,age&disease.)*&disease.(0)= &r sex &v/rl ;
ods output  ParameterEstimates=parameterestimates;
where bmi ne . and ment ne .;
run;

Proc print data= ParameterEstimates; run;


%mend;
%let data=&disease.1;
%adjustment(&disease.,resource,)
%adjustment(&disease.,resource, marital edu score employment mental)
%adjustment(&disease.,resource, marital edu score employment mental bmi)
%adjustment(&disease.,resource, marital edu score employment mental phy)
%adjustment(&disease.,resource, marital edu score employment mental alcohol)
%adjustment(&disease.,resource, marital edu score employment mental smoke)
%adjustment(&disease.,resource, marital edu score employment mental ment)
%adjustment(&disease.,resource, marital edu score employment mental bmi phy alcohol smoke ment)
*ods rtf close;

%macro interaction_age;

ods rtf file="P:\Analysis\Resources\SLOSH\results2\interaction_age_&disease..rtf";;

proc phreg data=&disease.1 fast ;
ods exclude none;
class resource(ref='1') wave(ref='4') mental(ref='0') sex(ref='2')  edu(ref='1') marital(ref='G') employment(ref='permanent') agegrp(ref='0');
model (ageb,age&disease.)*&disease.(0)= resource wave mental ageb resource*ageb  sex marital edu employment score/rl ;
run;
ods rtf close;

%mend;

%interaction_age;

%mend;

%cox(cvd)
%cox(chd)
%cox(cd)
%cox(mi)
%cox(is)
%cox(hs)
```

R codes

```
#All analyses repeatedly performed for subtypes of CVD.
##################check time-varying hazards############
aalen1_cvd<-aalen(Surv(ageb,agecvd,cvd==1)~resource+ageb+wave+sex+edu+employment+score+marital,data=CVD)
sink("P:/Analysis/Resources/FPS/Additional results/Results2_simulated/time-varying hazards.txt")
summary(aalen1_cvd)
sink()

pdf(file="P:/Analysis/Resources/FPS/Additional results/Results2_simulated/time-varying hazards.pdf",onefile=TRUE)
par(mar=c(2,2,2,2))
par(mfrow=c(4,4))
plot(aalen1_cvd,pointwise.ci=2)
dev.off()

#main effect
aalen2_cvd<-
aalen(Surv(ageb,agecvd,cvd==1)~const(resource)+const(wave)+const(sex)+const(edu)+const(employment)+const(score)+const(marital),data=CV
D)
aalen3_cvd<-
aalen(Surv(ageb,agecvd,cvd==1)~const(rankorg)+const(wave)+const(sex)+const(edu)+const(employment)+const(score)+const(marital),data=CVD)
aalen4_cvd<-
aalen(Surv(ageb,agecvd,cvd==1)~const(rankleader)+const(wave)+const(sex)+const(edu)+const(employment)+const(score)+const(marital),data=C
VD)
aalen5_cvd<-
aalen(Surv(ageb,agecvd,cvd==1)~const(support)+const(wave)+const(sex)+const(edu)+const(employment)+const(score)+const(marital),data=CVD)
aalen6_cvd<-
aalen(Surv(ageb,agecvd,cvd==1)~const(co)+const(wave)+const(sex)+const(edu)+const(employment)+const(score)+const(marital),data=CVD)
aalen7_cvd<-aalen(Surv(ageb,agecvd,cvd==1)~ const(rankorg)+ const(rankleader)+
const(support)+const(co)+const(wave)+const(sex)+const(edu)+const(employment)+const(score)+const(marital),data=CVD)


sink("P:/Analysis/Resources/FPS/Additional results/Results2_simulated/additive main.txt")
print(summary(aalen2_cvd))
print(summary(aalen3_cvd))
print(summary(aalen4_cvd))
print(summary(aalen5_cvd))
print(summary(aalen6_cvd))
print(summary(aalen7_cvd))

sink()


#################testing additive interaction: age, sex and edu############
#CVD
ra.cvd1<-
aalen(Surv(ageb,agecvd,cvd==1)~const(ra)+const(wave)+const(sex)+const(edu)+const(employment)+const(score)+const(marital),data=CVD)
ra.cvd2<-
aalen(Surv(ageb,agecvd,cvd==1)~const(resource):const(agegrp)+const(agegrp)+const(wave)+const(sex)+const(edu)+const(employment)+const(s
core)+const(marital),data=CVD)
ra.cvd3<-
aalen(Surv(ageb,agecvd,cvd==1)~const(resource)*const(agegrp)+const(wave)+const(sex)+const(edu)+const(employment)+const(score)+const(ma
rital),data=CVD)
ra.cvd4<-
aalen(Surv(ageb,agecvd,cvd==1)~const(resource)*const(ageb)+const(wave)+const(sex)+const(edu)+const(employment)+const(score)+const(marit
al),data=CVD)

sink("P:/Analysis/Resources/FPS/Additional results/Results2_simulated/cvd_ra.txt")
print("Joint effect")
print(summary(ra.cvd1))
print("Stratified result")
print(summary(ra.cvd2))
print("Global test")
print(summary(ra.cvd3))
print(wald.test(coef=ra.cvd3$gamma, Sigma=ra.cvd3$var.gamma, coef.null=c(15:18)))
print(summary(ra.cvd4))
print(wald.test(coef=ra.cvd4$gamma, Sigma=ra.cvd4$var.gamma, coef.null=c(15:18)))

sink()

sr.cvd1<-aalen(Surv(ageb,agecvd,cvd==1)~const(sr)+const(wave)+const(edu)+const(employment)+const(score)+const(marital),data=CVD)
sr.cvd2<-
aalen(Surv(ageb,agecvd,cvd==1)~const(resource):const(sex)+const(wave)+const(sex)+const(edu)+const(employment)+const(score)+const(marital
),data=CVD)
sr.cvd3<-
aalen(Surv(ageb,agecvd,cvd==1)~const(resource)*const(sex)+const(wave)+const(edu)+const(employment)+const(score)+const(marital),data=CVD
)
sink("P:/Analysis/Resources/FPS/Additional results/Results2_simulated/cvd_sr.txt")
print("Joint effect")
print(summary(sr.cvd1))
print("Stratified result")
print(summary(sr.cvd2))
```

```
print("Global test")
print(summary(sr.cvd3))
print(wald.test(coef=sr.cvd3$gamma, Sigma=sr.cvd3$var.gamma, coef.null=c(14:17)))
sink()

er.cvd1<-aalen(Surv(ageb,agecvd,cvd==1)~const(er)+const(sex)+const(wave)+const(employment)+const(score)+const(marital),data=CVD)
er.cvd2<-
aalen(Surv(ageb,agecvd,cvd==1)~const(resource):const(edu)+const(wave)+const(sex)+const(edu)+const(employment)+const(score)+const(marital
),data=CVD)
er.cvd3<-
aalen(Surv(ageb,agecvd,cvd==1)~const(resource)*const(edu)+const(wave)+const(sex)+const(employment)+const(score)+const(marital),data=CVD
)
sink("P:/Analysis/Resources/FPS/Additional results/Results2_simulated/cvd_er.txt")
print("Joint effect")
print(summary(er.cvd1))
print("Stratified result")
print(summary(er.cvd2))
print("Global test")
print(summary(er.cvd3))
print(wald.test(coef=er.cvd3$gamma, Sigma=er.cvd3$var.gamma, coef.null=c(14:21)))
sink()
```
